# Biochar from Pine Wood, Rice Husks and Iron-Eupatorium Shrubs for Remediation Applications: Surface Characterization and Experimental Tests for Trichloroethylene Removal

**DOI:** 10.3390/ma14071776

**Published:** 2021-04-03

**Authors:** Marta M. Rossi, Ludovica Silvani, Neda Amanat, Marco Petrangeli Papini

**Affiliations:** 1 Department of Chemistry, Sapienza University of Rome, P.le Aldo Moro 5, 00185 Rome, Italy; neda.amanat@uniroma1.it (N.A.); marco.petrangelipapini@uniroma1.it (M.P.P.); 2 Aedes S.r.l. Research and Development, Aedes Chimica e Ambiente, Via Cancelliera 12, 00041 Albano Laziale, Italy; ludovica.silvani@aedes.info

**Keywords:** biochar, adsorption, kinetic test, isotherm curves, fixed-bed test, groundwater remediation, chlorinated solvents

## Abstract

Nowadays porous materials from organic waste, i.e., Biochar (BC), are receiving increased attention for environmental applications. This study adds information on three BCs that have undergone a number of studies in recent years. A Biochar from pine wood, one from rice husk and one from *Eupatorium* shrubs enriched with Iron, labelled as PWBC, RHBC and EuFeBC respectively, are evaluated for Trichloroethylene (TCE) removal from aqueous solution. Physical-chemical description is performed by SEM-EDS and BET analysis. The decrease of TCE over time follows a pseudo-second order kinetics with increased removal by the PWBC. Freundlich and Langmuir models well fit equilibrium test data. The optimized values of the maximum adsorbed amount, q_max_ (mg g^−1^), follows this order 109.41 PWBC > 30.35 EuFeBC > 21.00 RHBC. Fixed-bed columns are also carried out. Best performance is again achieved by PWBC, which operates for a higher number of pore volume, followed by EuFeBC and RHBC. Continuous testing confirms batch studies and makes it possible to evaluate the workability of materials in configurations closer to reality. Results are promising for potential environmental application. In particular, the characterization of several classes of contaminants opens the doors to possible uses in mixed contamination cases.

## 1. Introduction

Halogenated hydrocarbons have been widely used as degreasers, process solvents and intermediates in various industrial sectors. As a result of mismanagement, use and disposal, these substances are very common pollutants in soil and groundwater. The distribution of Chlorinated Solvents, such as Perchloroethylene (PCE) and Trichloroethylene (TCE), in solid and liquid matrix of the subsoil is a process that depends not only on the chemical and physical characteristics, but also on the hydrological characteristics of the site. In general, many years after the primary source of contamination has been removed, chlorinated solvents act as persistent, slow-release sources in the saturated area at very low concentrations [1,2].

The oldest and most diffused system of groundwater treatment is the Pump and Treat, that uses wells for the extraction of the contaminated water and needs a successive treatment on external unit for the requalification of the liquid phase. In Italy, where PCE and TCE threshold limit (to be respected at the boundary of the site) are 1.1 and 1.5 µg L^−1^, many of the SIN (National Interest Sites) are still treated with this approach [3]. However, it has been known for decades that this strategy has a high environmental and economic impact linked to the energy demand due to the treatment of high volumes of liquid phase [4].

For the specific purpose of intercepting a contaminated plume, a widespread remediation technology involves the installation of Permeable Reactive Barriers (PRBs) [5]. Depending on the reactive material, the barrier removes the contaminant by chemical action (the most common PRBs are with Zero Valent Iron (ZVI)), by biological action or by adsorption (zeolites, activated carbon). Although Activated Carbon (AC) is recognized as the most preferred reactive material for its removal efficiency, it is also well known for the high costs associated with its use [6]. These days, the remediation fields are looking for more sustainable options [7], so low-cost materials are receiving much attention. The search for alternative adsorbing materials for environmental applications and the desire to reduce solid waste volumes explain the growing interest of the scientific community in Biochar (BC). BC is an ancient fertilizer derived from non-fossil-based carbonaceous materials such as agricultural and manure wastes [8]. According to some authors, it is produced to be applied to soil as fertilizer and its manufacture allows converting biomass into reusable products and energy [9,10,11,12,13]. Since the early decades of this century, the number of BC related papers has grown exponentially [14].

Recently, initiative like the International Biochar Initiative (IBI) and the European Biochar Certificate (EBC) are born to talk about BC and better define its standards, especially for the valorization of such residues in environmental field [15,16]. Most papers propose it as contaminants immobilizers due to its graphite-like structure, its porosity and surface area. BC from different feedstocks is investigated in several papers as possible adsorbent for the removal of heavy metals and organic contaminants from municipal wastewater as well as from industrial wastewater [17,18,19,20,21,22]. Furthermore, it is of particular interest to study these materials by also considering the interaction with subsoil’s bacteria. The favorable interaction between BC and microorganisms certainly could open the door to possible innovative applications as support for active biofilms for coupling adsorption and biodegradation strategies for environmental applications [23,24,25].

It is well known that many parameters influence the final characteristics of BC, which is why its production and surface alteration certainly select its final application [16,26,27,28,29]. When the pollutant is hydrophobic or aromatic, the aromatization of BC is a fundamental characteristic to increase the adsorption capability. By increasing the temperature of the heat treatment, the chemical structure of BC is constantly changing and eventually forms more graphitized carbon [30]. On the other hand, electrostatic interactions are responsible of ionic compounds immobilization, that is the reason why BC obtained at lower temperature should be a selective adsorbent for cationic species thanks to the presence of functional groups (e.g., phenol and carboxyl moieties) [31]. In addition, the size and structure of the pores are dependent on the composition of the raw materials and the temperature adopted during the heat treatment. To evaluate and predict the applicability of a material as an adsorbent, it is essential to combine textural characterization with batch and continuous adsorption tests.

In this work three different BCs are investigated: a pine wood BC (PWBC) obtained in a gasification unit in Germany [32]; a rice husk BC (RHBC), made in Indonesia using local technique [33] and a modified BC, the *Eupatorium adenophorum* Iron-enriched biochar (EuFeBC) from Nepal [34]. With respect to these BCs, certain studies have already been published for specific environmental purposes. PWBC showed promising results in the removal of Toluene from synthetic solutions [32] and it has been also demonstrated a greater capping efficiency of oil spill contaminated sediment, compared to a commercial AC [35]. More recently, RHBC has been reported to reduce the bioavailability of PCBs when utilized as amended in soil [36]. At the same time, the Iron enriched BC (EuFeBC) was firstly evaluated in a real soil contaminated by heavy metals [34], underlying that engineered BC should be considered for specific contamination situations [37].

Therefore, the three BCs were firstly characterized from a textural point of view (SEM-EDS analysis and BET analysis) as the adsorption of organic molecules depends on the available and suitable sites [38]; batch reactors (kinetic and equilibrium tests) were then performed to study the capacity of TCE removal from aqueous solution; finally, to simulate the conditions of a real configuration technology (i.e., PRB), fixed-bed reactors were performed. The majority of published literature characterizes Biochar by Batch tests. Nevertheless, Breakthrough curves obtained by continuous tests are useful to understand possible real application [39,40].

## 2. Materials and Methods

### 2.1. Biochars Description

**Figure 1:**
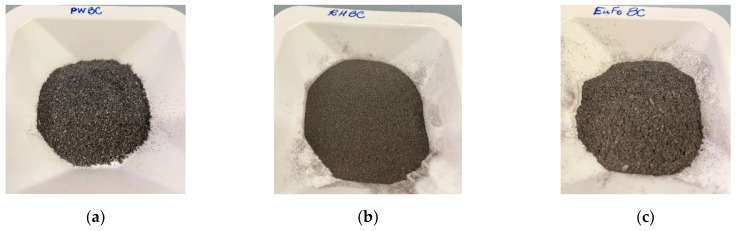
Photos of the investigated materials: (**a**) PWBC, (**b**) RHBC and (**c**) EuFeBC.

The materials were received thanks to previous collaboration between the La Sapienza University (Rome) and the University of Chemistry and Technology of Prague and the NGI (Norwegian Geotechnical Institute) of Oslo. The Pine Wood BC (PWBC) is obtained by gasifying wood at approximately 850 °C and using the resulting gases for energy recovering (wood gas generator V 3.90 Burkhardt and ECO 180 HG as heat and power plant, Plößberg bei Tirschenreuth, Germany). The technical data were extensively described elsewhere along with the composition of the resulting gas [32]. The Rice husk BC (RHBC) from rice (*Oryza sativa* L.) is produced with a low technology method consisting in husks pyrolysis in a little unit realized in Indonesia (kiln method). Conditions were 3.5 h pyrolysis duration and an average temperature of 300 °C [33]. In pre-treatment RHBC was sieved, so the only fraction < 125 µm was used. The third BC from *Eupatorium adenophorum* shrubs (EuFeBC) is obtained with Kon-Tiki flame curtain pyrolysis method, which leads back to the ancient methods of biomass combustion combined with a low gas emission. The process reaches approximatively 600 °C and powdered iron oxide-hydroxide (FeOOH) was added between fresh biomass layers. For additional details, see Silvani and Cornelissen work (2019) [34]. Since EuFeBC had a different particle size distribution, only a fraction less than 500 µm was selected and manually homogenized in a mortar. The materials for the experimental tests are shown in Figure 1.

### 2.2. SEM-EDS Acquisition and BET Analysis

To evaluate the morphology of the materials, a Zeiss Auriga (Germany) FESEM (Field Emission Scanning Electron Microscope) with Energy Dispersive Spectroscopy (EDS) has been used. BCs were dried in oven (Carbolite, Thetford, Norfolk, UK), 100 °C overnight, then the analysis was performed on untreated materials. Surface area, the Brunauer–Emmett–Teller (BET) method, and textural analysis were determined as described elsewhere [32].

### 2.3. Kinteric and Isotherm Tests

Kinetic and isothermal experiments were performed as described in Kastanek (2017) and co-workers [32].

For TCE adsorption tests, a mono-component solution was prepared in a Tedlar bag^®^ (Supelco, Bellefonte, PA, USA) (no headspace formation); a volume of pure TCE, (ACS ≥ 99.5%) Sigma-Aldrich^®^ (St. Louis, MO, USA), was spiked in order to achieve the decided concentration; the solution was horizontally shaken for one day. For the initial effective concentration, one volume of the contaminated solution was always sampled at the beginning of the test. The batch reactors (VWR International glass vials, Milan, Italy) were prepared by weighing a known amount of BC and fully filling with 0.02 L of the contaminated solution. The reactor was sealed by a Teflon butyl stopper (Wheaton, Millville, NJ, USA) and aluminum cap and mechanically shaken for the duration of the test.

For the kinetic study, the initial theoretical concentration (C_0_) was 100 mg L^−1^ with a solid/liquid ratio of 2.5 (in triplicate). Aliquots of the solution was sampled at 0.5, 1, 2, 3, 4, 5 and 24, 27 and 52 h in hermetically sealed glass vials sent to the analysis. The isothermal curve was designed to prepare the solution in a theoretical initial concentration range equal to 10–60–120 mg L^−1^, in contact with 0.010–0.020–0.050–0.100 g of BC. The solution was sampled at time 0 (C_0_), to assess the initial concentration, and at equilibrium time (C_e_). Based on the kinetic tests, 24 h was considered as enough time to ensure the achievement of equilibrium. Elemental analysis and isothermal curve were carried out on a commercial Norit AC, type Darco Sigma-Aldrich^®^, for comparison purpose only.

### 2.4. Column Experiments

**Figure 2:**
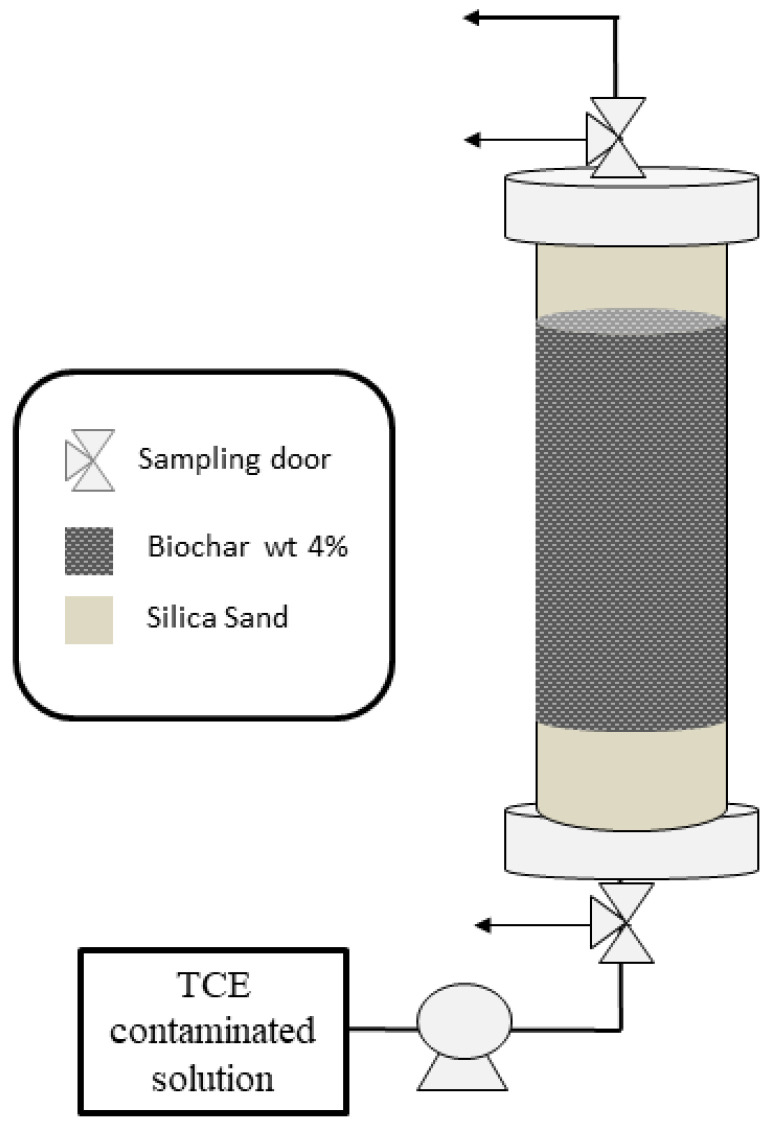
Experimental configuration of the fixed-bed column.

Three column tests were carried out to draw the breakthrough curves. Plexiglas (PMMA) columns (13 cm × 2.6 cm) were equipped with two sampling points, door IN and OUT, respectively at the entry and the exit of the column. The adsorbent materials were mixed with silica sand in quantities equal to 4 wt% (equivalents to about 2 g of reactive material). In detail, the first and the last 3 cm of the column were filled only with sand in order to make the liquid phase front homogeneous in the reactive zone (7 cm of bed height). A peristaltic pomp (Gilson Miniplus evolution) was used to pump the feeding solution upwards at 0.6 mL min^−1^ as flow rate. A tracer test with Br^−^ was first performed to obtain the residence time, the porosity and the pores volume. The feeding solution was approximately 5 mg L^−1^ of TCE. For TCE analysis, 1 mL of influent and effluent was sampled daily. Figure 2 illustrates a schematic draw of the experimental configuration. The tests were stopped when the outlet concentration (C_OUT_) became equal to that of the input (C_IN_), indicating that the bed adsorption capacity was exhausted.

### 2.5. Calculations

Kinetic data were fitted according to a pseudo-first order (PFO) model and pseudo-second order model (PSO) as described in [41,42]. The equations are written in Equations (1) and (2):

ln (q_e_ − q_t_) = ln q_e_ − k_1_t, (1)

t/q_t_ = (1/q_e_^2^ k_2_) + (1/q_e_) t. (2)

Removal efficiency %, after 24 h, is calculated as:

R_24_ (%) = (C_0_ − C_eq_)/C_0_ ∗ 100. (3)

The TCE adsorbed per gram of material at equilibrium, q_e_ and at the sampling time, q_t_ (mg g^−1^), are calculated using Equations (4) and (5). k_1_ (min^−1^) and k_2_ (mg g^−1^ min^−1^) are pseudo-first-order (PFO) and pseudo-second-order (PSO) adsorption rate constants. C_0_, C_e_ and C_t_ are respectively the TCE starting, the equilibrium concentration (24 h) and the concentration at the time of sampling (t), expressed in mg L^−1^, V is the volume of the solution (L). According to Equations (6) and (7), representing Langmuir and Freundlich models, the optimized parameters were obtained using Sigma Plot 12. q_max_ and K_L_ (L mg^−1^) are Langmuir coefficients; K_F_ (L g^−1^) and n are the Freundlich constants characteristics of the system [43].

q_e_ = (C_0_ − C_e_) V/w, (4)

q_t_ = (C_0_ − C_t_) V/w, (5)

q_e_ = q_max_ K_L_ C_e_/(1 + K_L_C_e_), (6)

q_e_ = K_F_ C_e_^n^. (7)

A three-parameter sigmoidal equation, Equation (8), was used to fit both tracer test data and breakthrough curves. A quantitative performance of the reactive bed has been determined. Some authors choose to define the breakthrough point once the compound can be detected in the effluent [40,44]. Alternatively, a C_OUT_/C_IN_ ratio can be defined in order to represent a tolerance limit (as a threshold concentration admitted by the local legislation) beyond which the effluent quality is no longer acceptable. In this case, the breakpoint was defined as the volume in which C_OUT_/C_IN_ = 0.05 and the bed saturation point was considered C_OUT_/C_IN_ = 0.95. The area between Y axis and breakthrough curve is proportional to TCE adsorbed by fixed-bed, hence the area under the curve was subtracted at the total area at the saturation point. Details on column data elaboration are presented in Supplementary Materials.

f = a/(1 + exp(x − x_0_/b)) (8)

### 2.6. Analytical Methods

TCE was determined with gas chromatography (GC) DANI MASTER, equipped with DANI 86.50 headspace auto-sampler ((DANI Instruments, Contone, Switzerland): capillary column (30 m × 0.53 mm ID × 3 um, TRB624) and a Flame Ionization Detector (FID) was used. The headspace analysis program was performed as follows: oven temperature 80 °C, manifold T 120 °C, transfer line temperature 180 °C, shaking softly for 1 min. The GC conditions were: He carrier gas (flow 10 mL min^−1^), 180 °C injector temperature split injection 1:2; 300 °C detector temperature with air, N_2_ and H_2_ for the FID (flows 240, 25, 60 mL min^−1^). The oven temperature was programmed as follows: 70 °C 0.5 min, 30 °C min^−1^ to 90 °C then 30 °C min^−1^ to 180 °C. For the quantitative determination of TCE, a calibration curve was obtained by dilution of a TCE/Ethanol stock solution in standards with concentration range 0.1–5 mg L^−1^.

Bromide was revealed with ion chromatograph Dionex (Sunnyvale, CA, USA) ICS-1000 IC with conductivity cell detector, equipped with Dionex AS-40 Auto sampler. Pre-column Dionex IonPac™ AG14 (4 × 50 mm) and a Dionex IonPac™ AS14 IC Column with suppressor AESR 500 4 mm (Thermo Fisher Scientific Chelmsford, MA, USA). The eluent phase was prepared with 3.5 mM Na_2_CO_3_ and 1.0 mM NaHCO_3_ with 1.2 mL min^−1^ as flow rate. The calibration curve was realized from 5 to 100 mg L^−1^.

## 3. Results and Discussion

### 3.1. Physicochemical Properties of Biochars

**Figure 3:**
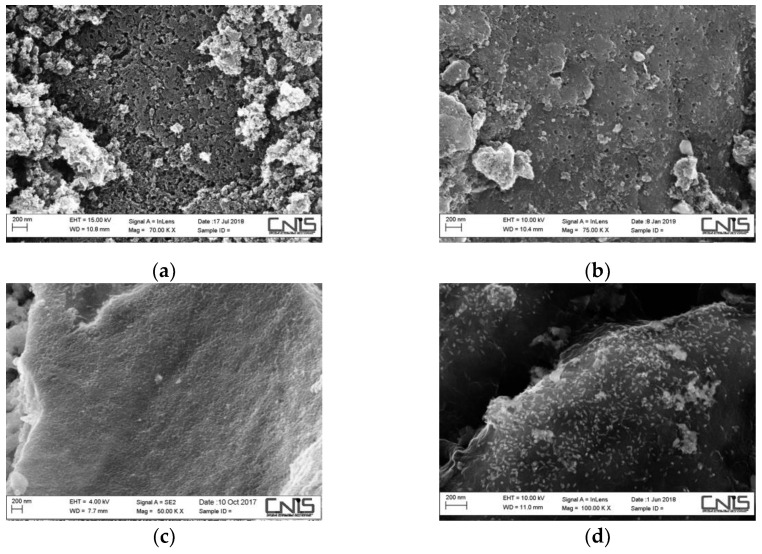
SEM images at 200 nm of resolution: (**a**) commercial Activated Carbon; (**b**) PWBC; (**c**) RHBC and (**d**) EuFeBC. Other images can be found in Supplementary Materials (Figures S1 and S2).

Physicochemical characteristics were studied using SEM-EDS. Here are reported the images obtained at resolution of 200 nm, also with the AC, the same used in Kastanek (2017) [32] (Figure 3). It is evident that there is a regular pore distribution on the PWBC surface, fairly similar to the AC. On the other hand, the other two materials prove to be very different and highlight the heterogeneity of the biomass materials that have not undergone any activation process.

Elemental analysis confirms the differences between all the BCs investigated due to the starting biomass and heat treatment. As expected, for PWBC the 95.84 wt% of the mass was C; 3.92 wt% O; 0.10 wt% S; 0.09 and 0.04 wt% of K and Mg (complete data in Figure S3). This is consistent with results concerning wood based biochar [45,46,47]. At high processing temperature, wood based feedstock may reach a carbon content greater than 95% and oxygen content less than 5% [8]. Traces of Mg, K, S, are commonly found in plants and soil. EuFeBC mostly consists of C (80.69 wt%) in the investigated area, with 13.15 wt% of O and Iron (1.17 wt%). The full list and elemental distribution maps are in S4. On the other hand, the percentage of Total Carbon in RHBC 41.24 and O/C ratio was higher [33]. These results confirm a relationship with the thermal treatment and high graphitization making PWBC the biochar closer to an activated carbonaceous material (elemental analysis of the commercial Activated Carbon in S5). Textural characterization of Pine Wood Biochar has already been defined with 343 ± 2 m^2^ g^−1^ and a total pores volume 0.383 cm^3^ g^−1^, mostly due to micro-pores, 0.136 cm^3^ g^−1^, comparable with the commercial AC [32]. BET method for surface area and pores distributions of sieved RHBC reports that the sample is micro/meso-porous with a specific surface area value of 81.0 ± 0.7 m^2^ g^−1^ of which 59 m^2^ g^−1^ are due to the micro-pores present. The total volume of pores is 0.101 cm^3^ g^−1^, including 0.0280 cm^3^ g^−1^ for the presence of micro-pores. The pore diameter is distributed between 0 and 800 Å, with a maximum of about 19.9 Å (in the mesoporous area studied). EuFeBC powder is mainly micro/meso-porous and has a specific surface area of 97 ± 1 m^2^ g^−1^, of which 81 m^2^ g^−1^ for micro-pores. The total pore volume is 0.0752 cm^3^ g^−1^, 0.0390 cm^3^ g^−1^ due to micro-pores. The pore diameter is distributed in the range 0 and 300 Å, with a maximum of around 19.8 Å in this area. Results are summarised in Table 1.

### 3.2. Adsorption Test in Batch Reactors

**Figure 4:**
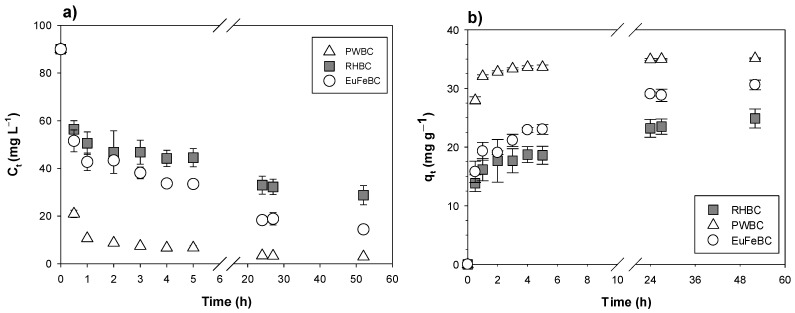
Kinetic results of TCE onto BCs. TCE Concentration (**a**) and TCE adsorbed per gram of BC (**b**) as a function of time. Error bars indicate the standard deviation.

The decrease in TCE concentration over time from C_0_ of approximately 90.07 mg L^−1^ can be seen in Figure 4a. Figure 4b illustrates the amount of TCE adsorbed per gram of biochar over time. q_t_ values have already increased after 30 min from the beginning of the test. The behavior of PWBC indicates a greater adsorbent capacity at the same contact time, compared to RHBC and EuFeBC. A greater adsorbent capacity of PWBC was calculated, with 33.6 ± 0.4 mg g^−1^; followed by 23.0 ± 0.83 mg g^−1^ for EuFeBC and 18.6 ± 1.5 mg g^−1^ for RHBC. 24 h were considered a sufficient time to determine the equilibrium mg TCE per gram of BC. More effective removal, based on Equation (3), was achieved by PWBC with 96.1 ± 0.4%, followed by EuFeBC and RHBC with 79.8 ± 4.2% and 63.8 ± 0.6% respectively. Rapid TCE removal and values of the mg g^−1^ achieved at the equilibrium time are similar with the literature, where BCs obtained at 300 °C and 700 °C (from soybean Stover and peanut shells) were compared [41]. It is also confirmed that the BCs produced at 700 °C were more effective, thus the pine wood biochar behavior is consistent with the literature. The parameters for both kinetic models are reported in Table 2. Because of the higher R^2^ values, PSO model works better for our kinetic data. According to some authors, a second order kinetic should represent a rate of adsorption controlled by direct interaction with solute and surface (i.e., chemisorption), as well as sharing or exchanging electron [26,41,43].

BC with better adsorption capacity has greater K_2_ value than the other two (following this order K_2_ PWBC > K_2_ RHBC > K_2_ EuFeBC). This demonstrates a greater affinity for TCE related to a faster binding. The q_e(cal)_ range values and both the k_1_ and k_2_ rate constants, are comparable with the literature. It is quite interesting to note that the k_2_ of PWBC is close to the one of Activated Carbon reported in the literature [41].

**Figure 5:**
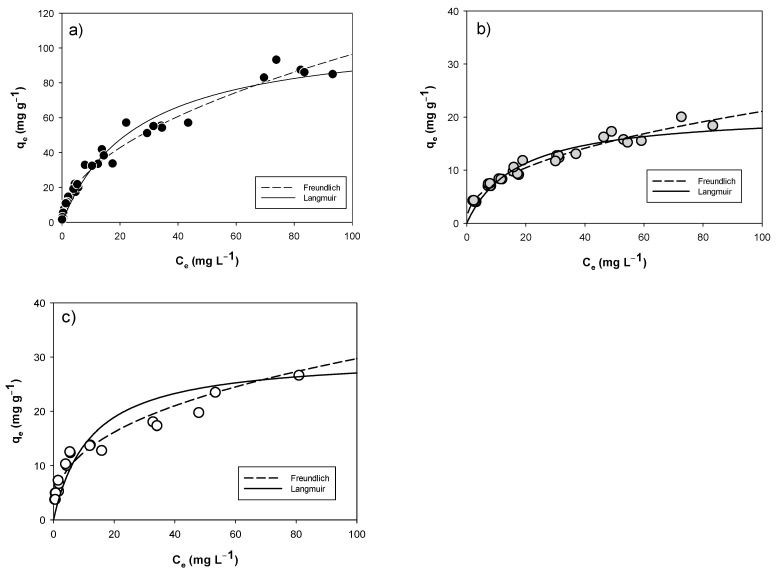
Isotherm curves of TCE onto investigated BCs: (**a**) PWBC, (**b**) RHBC and (**c**) EuFeBC.

Isothermal curves (Figure 5) follow the typical trend of the adsorption of a solute onto a solid material. Within the studied concentration range (C_e_), mg g^−1^ values increase strongly initially and then slow down as the concentration of Chlorinated Solvent increases (C_e_). In particular, the tested materials do not reach an obvious plateau at the maximum of C_e_ investigated, confirmed by a higher R^2^ of Freundlich model, compared to that of Langmuir.

The parameters obtained by nonlinear regression are given in Table 3, only to compare the performances. In addition, K_F_ and n values are higher for PWBC since the TCE removal and adsorption intensity are more evident [43].

In parallel, the maximum TCE adsorbent amount per gram of material (q_max_), calculated with Langmuir model, is absolutely greater for PWBC with 109.41 ± 5.62 mg g^−1^, while the q_max_ of EuFeBC and RHBC is 30.35 ± 2.49 and 21.00 ± 0.91 mg g^−1^, even though the Langmuir model has an R^2^ far from the unit. In S6 the isotherm curve of Activated Carbon and TCE is only reported for comparison purpose. The AC’s q_max_ values has the same order of magnitude (even if it is about 100 times that of PWBC), but it was obtained over a wider range of equilibrium concentrations.

Concerning the BC from pine wastes (PWBC) the parameters listed in Table 3 are comparable with results published by Ahmad Mahtab Lee and his colleagues towards TCE and pine needle derived BC at 700 °C [47] and with Freundlich parameters obtained for (Volatile Organic Compounds) VOCs sorption to peat moss BC investigated by Kim et al. (2017) [48]. More recently, A. Siggnins characterized and compared the behavior towards TCE removal of 15 BCs and a GAC. As verified in this work, BCs prepared at high temperatures (>600 °C) demonstrated a better performance [49].

The study was compared to the results of the batch tests performed with the same BC (PWBC) against Toluene. Values of q_max_ and K_L_ obtained for the TCE are of the same order of magnitude, although within a lower concentration range. Conversely, when comparing the Freundlich model parameters, n values are comparable whereas K_F_ values are higher for Toluene in synthetic seawater [32]. The only study looking at the possible adsorption capacity of RHBC was done by Silvani et al. (2019) with polychlorinated bisphenols (PCBs) contaminated soil. The results showed good performances of this BC in PCBs uptake, possibly related to its physical-chemical properties [36]. However, this is the first study with this Indonesian made biochar concerning the removal of a Chlorinated Solvent from an aqueous phase.

The performance of the BC enriched with Fe was attractive, as more than one removal mechanism may be implicated [34]. Recent papers have described the reduction of organic contaminants on the ZVI/BC magnetic surface [45,50,51,52]. In contrast, no intermediates of TCE reduction were revealed in the range of concentration investigated in our study. To compare the performance of the materials investigated, some basic characteristics (the starting biomass, the process temperature and the surface area (BET analysis)) are related to the maximum adsorption capacity, expressed by the isothermal parameter “q_max_” (optimized with the Langmuir adsorption model) (Table 4).

### 3.3. Column Tests

To simulate a real scenario of contamination, a low concentration of feeding solution was prepared (5 mg L^−1^ of TCE) and an intermediate flow rate was studied (0.6 mL min^−1^). The results of the tracer test (step signal) showed the typical behavior of a Plug Flow reactor (data shown in S7). For the three columns, the experimental hydraulic retention time were 58.2, 55.2, 55 min for PWBC, RHBC and EuFeBC (the operating conditions and geometrical characteristics were the same for all the columns). The estimated porosity was 50%, 47% and 48%, in the same order. Dispersion phenomena are probably responsible of little deviations in the shape of the curves. This is a proof of a difference in the fixed-bed packing due to several particle sizes of the BCs and the manual preparation of the columns itself.

**Figure 6:**
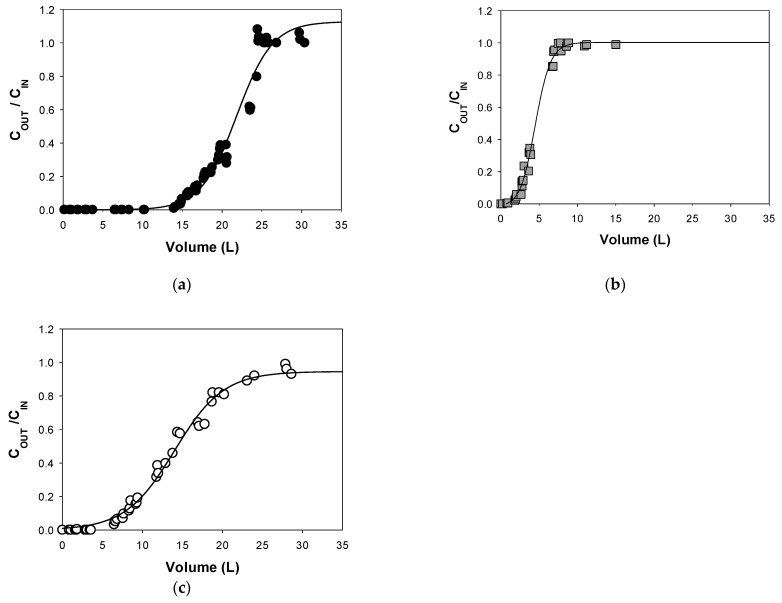
Breakthrough curves of TCE onto (**a**) PWBC; (**b**) RHBC and (**c**) EuFeBC. Fixed-bed reactors treated about 0.8 L die^−1^ and about 25 pore volume per day.

The breakthrough curves are shown in Figure 6. The concentration at the outlet normalized with that in the inlet (C_OUT_/C_IN_) is reported as a function of the volume of contaminated water treated (L). By comparing the experimental data, the first breakthrough point (C_OUT_/C_IN_ = 0.05) is reached by the RHBC bed at 2.04 L, corresponding to 2.5 days of working and 65 pore volumes. The EuFeBC’s breakthrough was revealed at 6.5 L (7.5 days and about 195 numbers of pore volume) and finally, after 17 days of continuous feeding and 408 number of pore volumes, also PWBC’s column reached the breakthrough point at 14.8 L, indicating that the BC from pine wood has treated higher volumes of contaminated water.

The curves diverged from the conventional sharp sigmoid probably because of the heterogeneity of the particle size. Diffusion phenomena may occur because a steeper profile is most often checked when a small particle moves into a homogeneous reactive bed. [39]. This is more evident in the EuFeBC curve, where the adsorbent bed consists of grain particles of different size (the fraction less than 500 µm, manually homogenized, was used).

Since the mass of contaminant adsorbed by the reactive zone is proportional to the area to the left of the curve, from the difference between the total area and the area under the curves the practical capacity (mg g^−1^) for each BC fixed-bed column was calculated. PWBC has removed more mg of TCE per grams (16.6 mg g^−1^), followed by EuFeBC (5.77 mg g^−1^) and RHBC (2.23 mg g^−1^), confirming the hypothesis made from characterization and batch tests. Furthermore, the expected adsorption capacity of the thermodynamic study was confirmed. 17.4, 8.85 and 4.73 mg g^−1^ were calculated with Langmuir parameters, choosing C_IN_ = 5 mg L^−1^ as the equilibrium concentration (summarized in Table S1).

## 4. Conclusions

This study highlights the potential use of Biochar as an adsorbent in environmental contexts, especially because “activation processes” are not required. Moreover, results have increased the acknowledge on three BCs recently investigated as a possible low-cost adsorbent of a wide range of ubiquitous contaminants and their effectiveness in different contaminated matrices (e.g., soil and groundwater).

In this respect, the heat treatment temperature (850–600–300 °C) must have affected the specific surface area. The materials are mainly meso-micro porous, and all the three BCs have pores diameters in the appropriate range for TCE adsorption (0–80 nm).

From a real application point of view, PWBC and EuFeBC seem to have better performance due to the sharp increase of the isotherm curves at very low C_e_. This condition is indeed more representative of a real contaminated site indeed, particularly in the case of an aging contaminated situation or in a Chlorinated Solvent plume. With regard to the pollutant adsorption, effectiveness depends on how the maximum mg g^−1^ at very low concentration is reached combined with a fast-kinetic removal. Our results are in line with the literature, confirming higher parameter values for higher temperature BC (PWBC), both for the kinetic and thermodynamic models. Moreover, column tests allowed to evaluate the performance of the materials for a high number of pore volumes, demonstrating the possibility to treat high volumes of water for long periods if compared to real conditions (lower ground water velocity and low concentrations of the contaminant).

The RHBC is still interesting as a cost-effective BC produced by a low-technology method and more investigations should be performed towards hydrophilic contaminants such as heavy metals. Finally, the hopeful results obtained by the engineered *Eupatorium adenophorum* BC allow expansion of the possibilities of use in case of mixed contamination (co-presence of organic solvents and heavy metals) thanks to the Iron active sites. A classification of the BC based on the starting biomass and the process conditions, together with a quality control of the final product, could make this solid waste an economically competitive material.

## Figures and Tables

**Table 1:** Results of textural characterization.

Material	Specific Surface Area (m^2^ g^−1^)	Total Pores Volume (cm^3^ g^−1^)	Micro-Pores Volume (cm^3^ g^−1^)
PWBC ^1^	343 ± 2	0.383	0.136
RHBC	81.0 ± 0.7	0.101	0.028
EuFeBC	97 ± 1	0.075	0.039
Commercial AC ^1^	712 ± 2	0.736	0.281

^1^ Results reported by Kastanek et al. (2027) [32].

**Table 2:** Kinetic parameters for TCE on tested BCs.

Material	Pseudo-First Order			Pseudo-Second Order		
	q_e(cal)_ (mg g^−1^)	K_1_ (min^−1^)	R^2^	q_e(cal.)_ (mg g^−1^)	K_2_ (g (mg min^−1^))	R^2^
PWBC	0.16	0.029	0.83	34.36	0.0053	0.99
RHBC	1.68	0.0072	0.89	19.42	0.0041	0.99
EuFeBC	2.28	0.0067	0.92	24.44	0.0019	0.99

**Table 3:** Optimized parameters of Freundlich and Langmuir models.

Material	Freundlich			Langmuir		
	n	K_F_ (L g^−1^)	R^2^	q_max_ (mg g^−1^)	K_L_ (L mg^−1^))	R^2^
PWBC	0.50 ± 1.6 × 10^−2^	9.44 ± 0.60	0.98	109.41 ± 5.62	3.8 × 10^−2^ ± 4.8 × 10^−3^	0.96
RHBC	0.43 ± 1.4 × 10^−2^	2.83 ± 0.14	0.97	21.00 ± 0.91	5.8 × 10^−2^ ± 6.3 × 10^−3^	0.93
EuFeBC	0.37 ± 1.8 × 10^−2^	5.21 ± 0.38	0.96	30.35 ± 2.49	8.2 × 10^−2^ ± 2.2 × 10^−2^	0.85

**Table 4:** Characteristics of various Biochar and commercial Activated Carbon studied and the maximum TCE adsorbed per grams of material (according to the Langmuir model).

Material Type	Peak Temperature (°C)	Surface Area (m^2^ g^−1^)	q_max_ (mg g^−1^)	Range of C_e_ (mg L^−1^)	Reference
Pine Wood BC	850	343 ± 2	109.41 ± 5.62	10–120	Kastanek et al., 2017 [32]; this work
Rice husks BC	300	81.0 ± 0.7	21.00 ± 0.91		
*Eupatorium shrubs* BC + Fe(OOH)	600	97 ± 1	30.35 ± 2.49		
Norit AC Darco^®^	-	712 ± 2	260.33 ± 22.32	10–200	
Herbal Pomace BC	600	374	562.157	200	Siggins et al., 2020 [49]
Oak BC	650–720	216	22.093		
Spruce BC	600	39	46.754		
GAC Darco^®^	-	633	90.680		
Soybean Stover BC	300	5.61	12.48	2–20	Ahmad et al., 2012 [53]
Soybean Stover BC	700	420.3	31.74		
Peatnut Shell BC	300	3.14	12.12		
Peatnut Shell BC	700	448.2	32.02		
AC Darco^®^ G-60	-	1110	50.01		
Pine Needle BC	300	4.09	205.417	5–50	Ahmad et al., 2013 [47]
Pine Needle	500	13.06	320.251		
Pine Needle	700	390.52	211.189		

## Data Availability

Data sharing not applicable. No new data were created or analyzed in this study. Data sharing is not applicable to this article.

## Supplementary Materials

The following are available online at https://www.mdpi.com/article/10.3390/ma14071776/s1, Figure S1: Other SEM images of Rice Husk biochar at 200 µm, 100 µm, and 1 µm, Figure S2: Other SEM images with a backscatter electron detector (BSD) of Eupatorium Iron enriched Biochar (Silvani et al. 2019) at 100 µm, 20 µm, 10 µm. It is emphasized the presence of well-defined and bright patches, probably caused by the high elemental weight, Iron (Lawrinenko et al. 2017), Figure S3: Pine Wood Biochar’s EDX spectra, and results of two different areas (indicated by PWB_1 and PWB_2 in the following photo) confirm the high presence of carbon (95.84 and 91.88 %wt C) concerning other elements due to the high thermal treatment, Figure S4: EDX data of a selected area of Eupatorium Iron enriched biochar and elemental distribution maps, Figure S5: EDS data of a selected zone of the Norit Activated Carbon Sigma Aldrich^®^. As reported in Figure S3, this composition is very close to the biochar obtained by the wood-based biomass, Figure S6: Isotherm curve of TCE on Activated Carbon Norit Sigma Aldrich^®^ as reference was realized only with a comparison purpose, Figure S7: Column tests data elaboration. The panel shows the step signal tracer test curves of the three columns packed with the investigated biochars, Table S1: Some parameters and quantitative performance of fixed-bed biochars columns.
