# Supplementary material for: Biochar from Pine Wood, Rice Husks and Iron-Eupatorium Shrubs for Remediation Applications: Surface Characterization and Experimental Tests for Trichloroethylene Removal
Source: Materials (Basel). 2021 Apr 3;14(7):1776. doi: 10.3390/ma14071776 (PMC8038453; doi:10.3390/ma14071776)
Supplement: Supplementary file 1 [file materials-14-01776-s001.pdf]

materials

MDPI

Supplementary Materials

Biochar from Pine Wood, Rice Husks and Iron-Eupatorium Shrubs for Remediation Applications: Surface Characterization and Experimental Tests for Trichloroethylene Removal

Marta M. Rossi [1,*], Ludovica Silvani [2], Neda Amanat [1] and Marco Petrangeli Papini [1]

[1] Department of Chemistry, Sapienza University of Rome, P.le Aldo Moro 5, 00185 Rome, Italy; neda.amanat@uniroma1.it; marco.petrangelipapini@uniroma1.it
[2] Aedes S.r.l., Research and Development, Via Cancelliera 12, 00041 Albano Laziale, Rome, Italy; ludovica.silvani@aedes.info
* Correspondence: martamaria.rossi@uniroma1.it

Supporting information consists of 6 text sections, Figures and Tables S1, S2, S3, S4, S5 and S6.

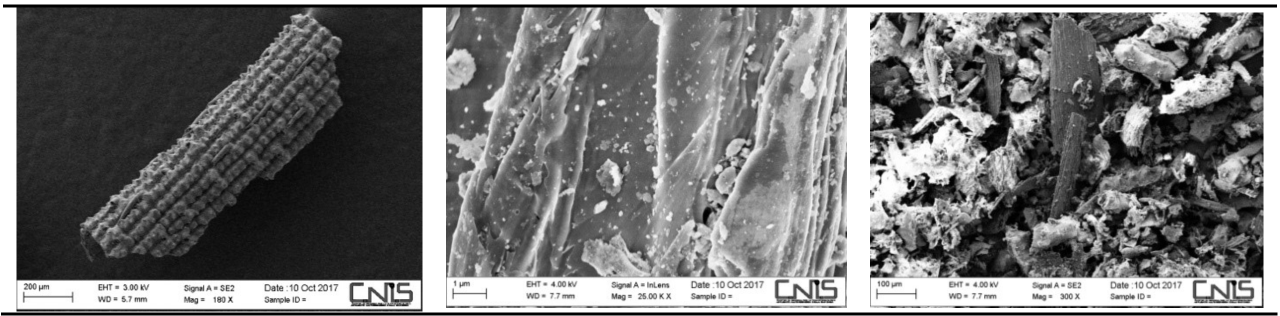

Figure S1. Other SEM images of Rice Husk biochar at 200 μm, 100 μm, and 1 μm.

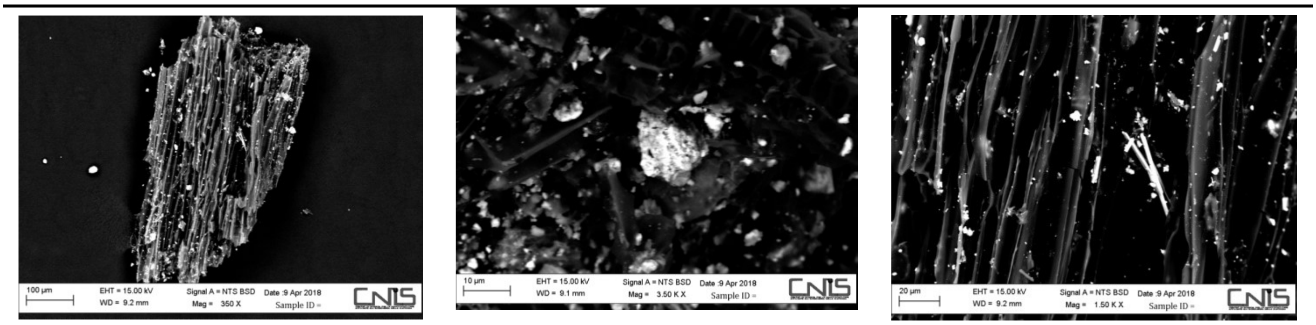

Figure S2. Other SEM images with a backscatter electron detector (BSD) of Eupatorium Iron enriched Biochar [1] at 100 μm, 20 μm, 10 μm. It emphasizes the presence of well-defined and bright patches, probably caused by the high elemental weight, iron [2].

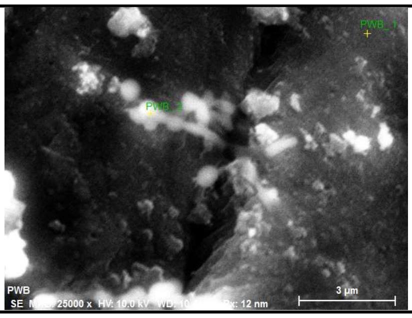

PWB_1	PWB_2

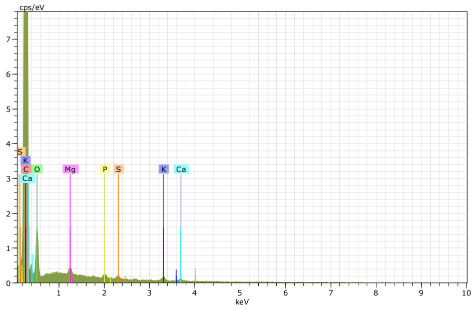

Spectrum: PWB_ 1

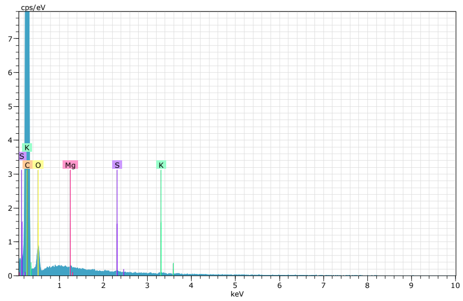

Spectrum: PWB_ 2

PWB_1

El	AN	Series	unn. C [wt.%]	norm. C [wt.%]	Atom. C [at.%]	Error (1 Sigma) [wt.%]
C	6	K-series	95.84	95.84	96.94	10.48
O	8	K-series	3.92	3.92	2.98	0.71
S	16	K-series	0.10	0.10	0.04	0.03
K	19	K-series	0.09	0.09	0.03	0.03
Mg	12	K-series	0.04	0.04	0.02	0.03
Total:			100.00	100.00	100.00	

PWB_2

El	AN	Series	unn. C [wt.%]	norm. C [wt.%]	Atom. C [at.%]	Error (1 Sigma) [wt.%]
C	6	K-series	91.88	91.88	94.33	10.08
O	8	K-series	6.66	6.66	5.14	1.07
K	19	K-series	0.48	0.48	0.15	0.05
Mg	12	K-series	0.22	0.22	0.11	0.04
Ca	20	K-series	0.33	0.33	0.10	0.05
S	16	K-series	0.22	0.22	0.08	0.04
P	15	K-series	0.20	0.20	0.08	0.04
Total:			100.00	100.00	100.00	

Figure S3. Pine Wood Biochar's EDX spectra and results of two different areas (indicated by PWB_1 and PWB_2 in the following photo) confirm the high presence of carbon (95.84 and 91.88 %wt C) concerning other elements due to the high thermal treatment [3–7].

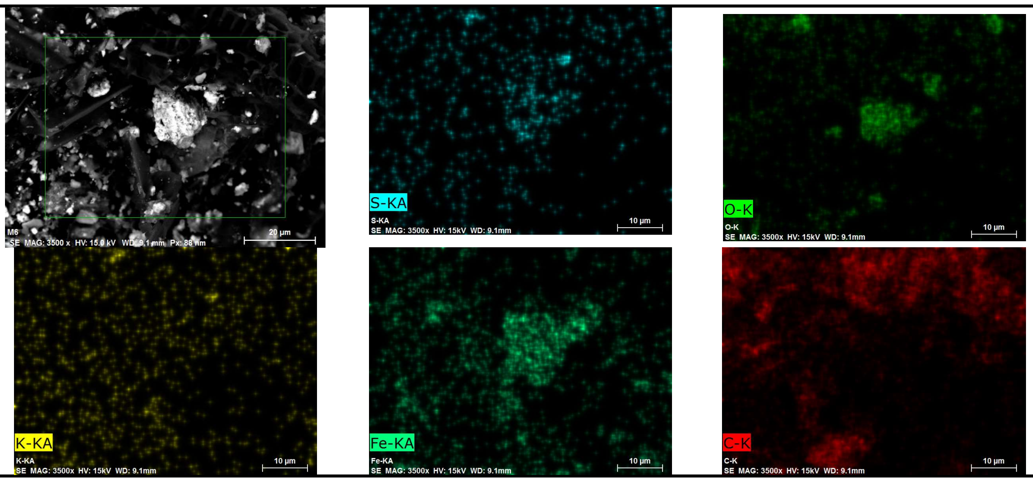

Spectrum: Roma17B41_30 keV_2.5 kX_60 um_ 63 90

El	AN	Series	unn. C [wt.%]	norm. C [wt.%]	Atom. C [at.%]	Error (1 Sigma) [wt.%]
**C**	6	K-series	80.69	80.69	87.22	10.47
**O**	8	K-series	13.15	13.15	10.67	2.91
**K**	19	K-series	2.35	2.35	0.78	0.13
**S**	16	K-series	1.06	1.06	0.43	0.08
**Ca**	20	K-series	0.85	0.85	0.27	0.08
**Fe**	26	K-series	1.17	1.17	0.27	0.14
**Mg**	12	K-series	0.24	0.24	0.13	0.05
**P**	15	K-series	0.19	0.19	0.08	0.04
**Si**	14	K-series	0.15	0.15	0.07	0.04
**Al**	13	K-series	0.15	0.15	0.07	0.04

Total: 100.00 100.00 100.00

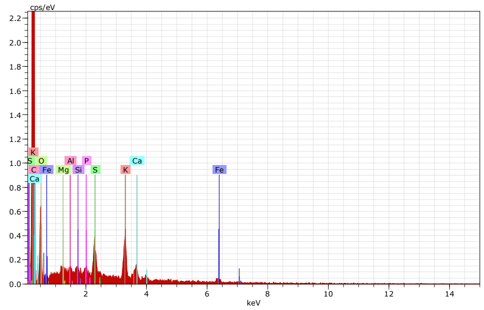

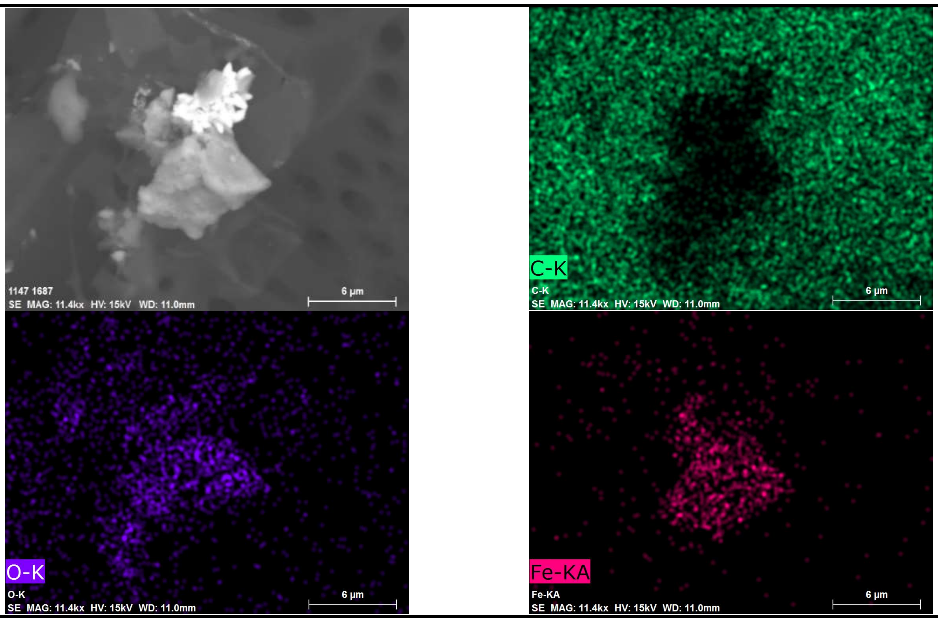

Figure S4: EDX data of a selected area of Eupatorium Iron enriched biochar and elemental distribution maps. The presence of Iron Oxo-Hydroxide particles is confirmed by the images of the element distribution mapping of the three zones investigated. Oxygen and iron are overlapped.

Spectrum: Activated Carbon_15 keV_5 KX_60 um_ 63 35

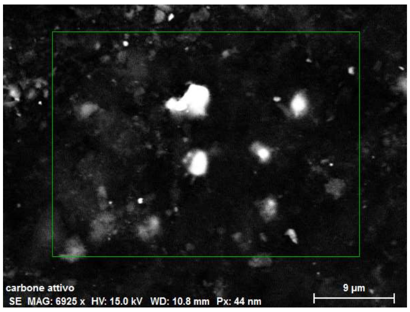

El	AN	Series	unn. C [wt.%]	norm. C [wt.%]	Atom. C [at.%]	Error (1 Sigma) [wt.%]
C	6	K-series	91.49	91.49	94.20	10.72
O	8	K-series	6.44	6.44	4.97	1.39
S	16	K-series	0.86	0.86	0.33	0.07
Si	14	K-series	0.68	0.68	0.30	0.06
Na	11	K-series	0.19	0.19	0.10	0.04
Ti	22	K-series	0.35	0.35	0.09	0.05

Total: 100.00 100.00 100.00

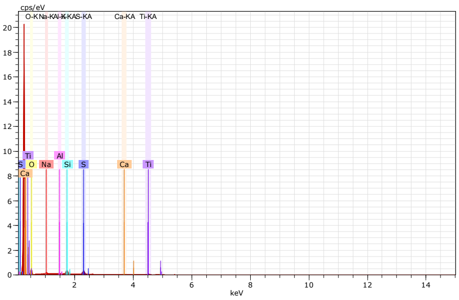

Figure S5: EDS data of a selected zone of the Norit Activated Carbon Sigma Aldrich®. As reported in S3 this composition is very close to the biochar obtained by the wood-based biomass.

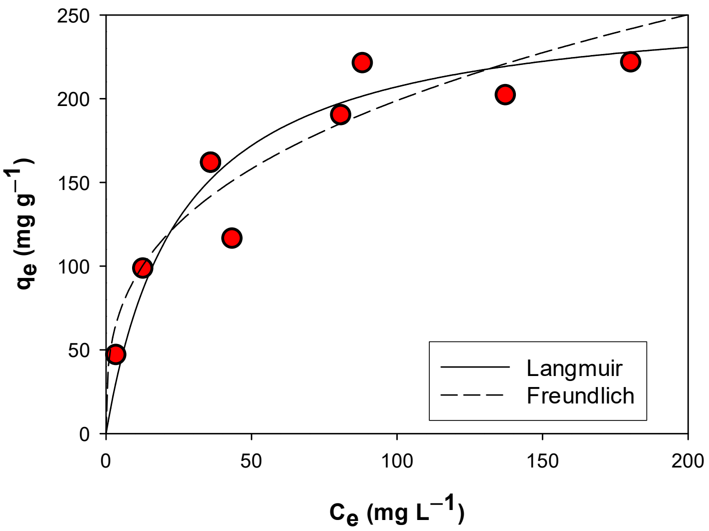

Figure S6: Isotherm curve of TCE on Activated Carbon Norit Sigma Aldrich® as reference was only realized for comparison purposes**.** Parameters obtained by non-linear regression models are q_{max} (mg g^{-1}) 260.33 ± 22.31 and K_L (L mg^{-1}) 0.038 ± 0.012, from the Langmuir model, and 0.88 as. K_F (L g^{-1}) 43.31 ± 10.47, n 0.33 ± 0.052 and 0.88 R^2.

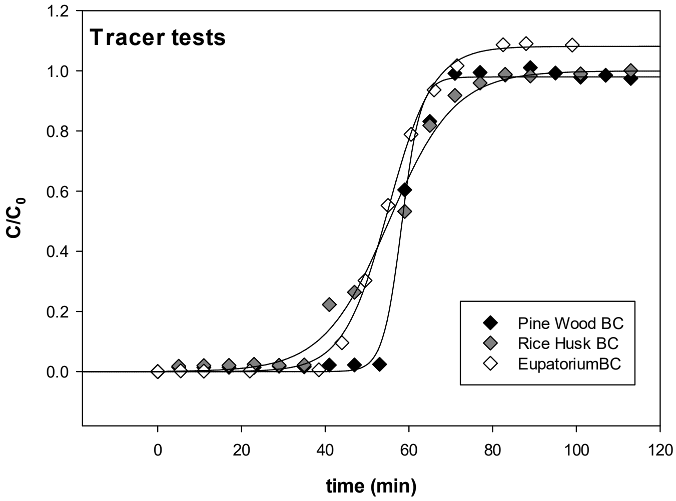

Figure S7. Column tests data elaboration. The panel shows the step signal tracer test curves of the three columns packed with the investigated biochar's.

Tracer test allowed us to determine the volume of pores (the volume occupied by the mobile phase) and the presence of stagnant zones or preferential paths, possible inconveniences due to constitutional defects in the column, or non-homogeneous packing of the porous medium. Knowing the flow rate (Q) and the residence time (herein expressed as t_0), the pores volume is given by the following relationship (Equation (S1)).

$$v_p = t_0 Q \tag{S1}$$

where:
v_p is the volume of pores;
Q is the flow rate.
t_0 is the residence time.
Pores Volume allows to calculate the porosity of the solid phase (Equation (S2)):

$$\vartheta = \frac{v_p}{v_c} \quad \text{(S2)}$$

ϑ is the porosity and v_c is the geometrical volume of the column. Porosity is representative of the space available to the mobile phase between the interstices of the grain of the material and the intragranular porosity. The average pore speed is given by Equation (S3):

$$u = \frac{L}{t_0} \quad \text{(S3)}$$

In this case, the residence time is calculated by the x_0 of the three parameters Sigmoidal function (Equation 8) optimized on experimental data. Pore volume is derived from the multiplication of the residential time with flow rate (0.6 ml min^{-1}); then the ratio between pores volume and geometric volume (68.98cm^3) is the porosity (%).

Table S1. Some parameters and quantitative performance of fixed-bed biochar's columns. The breakthrough point was defined at the volume where $C_{OUT}/C_{IN} = 0.05$. On the other hand, the saturation point was considered at $C_{OUT}/C_{IN} = 0.95$. The area between the Y-axis and breakthrough curve is proportional to the amount of TCE adsorbed; hence, the area under the curve was subtracted at the total area at the saturation point. mg TCE g^{-1} obtained by columns' data elaboration are comparable with values obtained by the calculation using batch models (considering 5 mg L^{-1} as equilibrium concentration).

Materials	C_{IN} (mg L^{-1})	Flow Rate (ml min^{-1})	x_0(min)	Porosity (%)	Area Under curve	Total Area	mgTCE g^{-1} Column	mgTCE g^{-1} Batch Model
**PWBC**			58.2	50	14.8	23.3	16.6	17.4
**RHBC**	5	0.6	55.2	47	5.4	7.6	2.2	4.7
**EuFeBC**			55.1	48	19.8	23.7	5.7	8.9

References

1. Silvani, L. *et al.* Can biochar and designer biochar be used to remediate per- and polyfluorinated alkyl substances (PFAS) and lead and antimony contaminated soils? *Sci. Total Environ.* **694**, 133693 (2019).
2. Lawrinenko, M. *et al.* Macroporous carbon supported zerovalent iron for remediation of trichloroethylene. *ACS Sustain. Chem. Eng.* **5**, 1586–1593 (2017).
3. Kastanek, P. *et al.* Characterizing Biochar as Alternative Sorbent for Oil Spill Remediation. *Sci. Rep.* **7**, 1–10 (2017).
4. Kameyama, K., Miyamoto, T. & Iwata, Y. The preliminary study of water-retention related properties of biochar produced from various feedstock at different pyrolysis temperatures. *Materials (Basel).* **12**, (2019).
5. Zhang, M. *et al.* Adsorptive Removal of Trichloroethylene in Water by Crop Residue Biochars Pyrolyzed at Contrasting Temperatures: Continuous Fixed-Bed Experiments. *J. Chem.* **2015**, (2015).
6. Ahmad, M. *et al.* Trichloroethylene adsorption by pine needle biochars produced at various pyrolysis temperatures. *Bioresour. Technol.* **143**, 615–622 (2013).
7. Ai, J. *et al.* Biochar Catalyzed Dechlorination - Which Biochar Properties Matter? *J. Hazard. Mater.* **406**, 124724 (2020).
